# Preliminary estimation of risk factors for admission to intensive care units and for death in patients infected with A(H1N1)2009 influenza virus, France, 2009-2010

**DOI:** 10.1371/currents.RRN1150

**Published:** 2010-03-09

**Authors:** Thomas Hanslik, Pierre-Yves Boelle, Antoine Flahault

**Affiliations:** ^*^Université Versailles Saint Quentin; ^†^INSERM U707; Faculté de Médecine Pierre et Marie Curie; Paris; France and ^‡^EHESP School of Public Health

## Abstract

To estimate the magnitude of the risks associated with age, obesity, pregnancy and diabetes, we compared the prevalence of these conditions reported in hospitalized severe cases to that in the general population, during the 2009-2010 A(H1N1) pandemic flu in France. Pregnancy, obesity, heart failure and diabetes were risk factors for admission into an intensive care unit (OR=5.2 [95%CI 4.0-6.9], 3.8 [3.0-4.9], 3.3 [2.6-4.1] and 2.8 [2.3-3.4], respectively). Only heart failure, obesity, and diabetes were significantly associated with death (OR=6.9 [4.9-9.8], 3.6 [1.9-6.2], and 3.5 [2.5-5.1], respectively). Elderly adults were at lower risk of being admitted into an ICU, but at higher risk of death.

The last pandemic flu has been characterized by a high rate of hospitalization for severe cases [1]. It has been reported that there is a surprisingly high rate of obese or pregnant persons among the hospitalized patients. However, the risk of such conditions has not been quantified yet. 

To estimate the magnitude of the risks associated with age, obesity, pregnancy and diabetes, we compared the prevalence of these conditions reported in hospitalized severe cases to that in the general population, using the already available databases.

## Methods

In France, the A(H1N1)v influenza epidemic started in week 37 of 2009 and lasted until week 1 of 2010. Since November 2009, all cases hospitalized in intensive care units (ICU) have been reported to the National Institute for Public Health Surveillance (Institut de Veille Sanitaire, InVS). The descriptive data published in the InVS weekly bulletin [2] were used to estimate prevalence of obesity, diabetes and pregnancy in severe cases hospitalized in metropolitan France.

The following data sources in the general population were used:

- Age and gender distribution in the general population were obtained from the National Institute of Statistics and Economic Studies [3].
- Pregnancy prevalence in the French general population was derived from birth incidence in France. As age class for the influenza population was predetermined, we used the available age class including child-bearing-aged women (i.e. the 15-64 years age class) as a denominator to estimate prevalence of pregnancy.
- Diabetes prevalence in the general French population was estimated in 2007 [4].
- Obesity prevalence in France was estimated in 2006 for those 3-17 years old [5], and in 2009, for those 18 and older [6]. From these data, we derived the overall prevalence of obesity in France. Once again, as age class for the influenza population was predetermined, we ascribed the total number of obese cases to the >1 year age class. 
- Heart failure prevalence has been estimated in patients aged 60 years and more seen in general practice in France. Prevalence in the general population was derived from this study [7].

Yates' corrected Chi2 were used to compare prevalence.

## Results

Results are reported in the table. Among the conditions studied, pregnancy, obesity, heart failure and diabetes were risk factors for admission into an ICU (OR=5.2 [95%CI 4.0-6.9], 3.8 [3.0-4.9], 3.3 [2.6-4.1] and 2.8 [2.3-3.4], respectively). Only heart failure, obesity and diabetes were significantly associated with death (OR=6.9 [4.9-9.8], 3.6 [1.9-6.2], and 3.5 [2.5-5.1], respectively). Compared to elderly subjects, infants, young and middle-aged adults were at increasing risk of being admitted into an ICU. Children, adolescents, young, and middle-aged adults were at decreasing risk of death.

**Figure:**
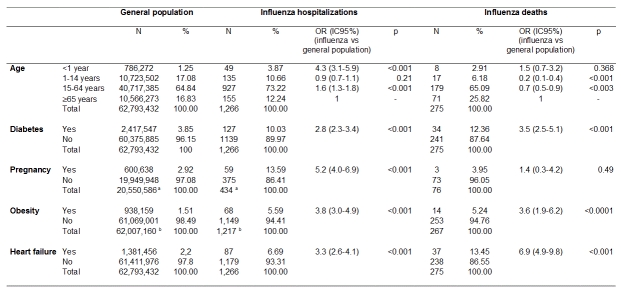
Age and gender distributions and diabetes, obesity and pregnancy prevalence in the French general population and in persons hospitalized in an intensive care unit for severe influenza A(H1N1)v infection( ^a^ Total number of women in the 15-64 years age class ; ^b^ Total number of persons aged 1 year and more)

## Discussion

These estimates show that pregnancy and age < 1 year are the strongest risk factors for admission into an ICU following influenza A(H1N1)v infection, but are not associated with increased risk of death. On the contrary, heart failure diabetes and obesity clearly increase the risk of being a severe case and dying. Elderly adults were at lower risk of being admitted into an ICU, but at higher risk of death.

There are some limitations to these estimates. Risk factors estimates from case/control studies must be assessed for biases, confounding and precision.

We used the whole population as control. This means that the reported estimates are not conditional to influenza infection, but rather measure the risk of both infection and severity or death.

The risk factors reported here may not be independent, as the results were not adjusted for we could not break down control characteristics with sufficient detail. For example, diabetes is reportedly up to seven times more prevalent in obese persons [6], so that most diabetes cases were likely among obese patients. The larger odds-ratio for diabetes for hospitalization suggests an additional risk compared to obesity alone, however this was not found for death. A specific control sample is required to allow for more precise adjustments. The interdependency of other risk factors is less clear. For example, if obesity was confounded by age, one would expect age to be a monotonously increasing risk factor as the prevalence of obesity increases with age, and this was not the case.

We assumed that there was no uncertainty in prevalence for controls. While this may be almost true for age, sex and pregnancies, it is less reasonable for diabetes and obesity which were measured on samples. This may lead to too short confidence intervals. However, the variance of the estimated odds-ratios is mostly due to the limited number of influenza severe cases and deaths, so that the impact should be limited.Finally, we did not have a precise age distribution of severe cases, leading to approximations of the selected age groups for comparison with the general population.    The estimates of risk for severe cases of influenza A(H1N1)v infection will have to be analyzed more precisely. They can, however, help devise vaccination strategies and enhance antiviral use recommendations.

## Acknowledgement

Jacques Cheymol, MD, for searching populational obesity data. Ilana Levin, MPH, for editorial support.

## Funding information

Public sources of funding from Inserm and EHESP.

## Competing interests

The authors have declared that no competing interests exist.

## References

[ref-33175069] ANZIC Influenza Investigators, Webb SA, Pettilä V, Seppelt I, Bellomo R, Bailey M, Cooper DJ, Cretikos M, Davies AR, Finfer S, Harrigan PW, Hart GK, Howe B, Iredell JR, McArthur C, Mitchell I, Morrison S, Nichol AD, Paterson DL, Peake S, Richards B, Stephens D, Turner A, Yung M. Critical care services and 2009 H1N1 influenza in Australia and New Zealand. N Engl J Med. 2009 Nov 12;361(20):1925-34. 10.1056/NEJMoa0908481 19815860

[ref-1214399615] Institut de Veille Sanitaire. http://www.invs.sante.fr/surveillance/grippe_dossier/points_h1n1/grippe_A_h1n1_260110/Bulletin_grippe_26_01_10.pdf

[ref-397635343] Institut national de la statistique et des études économiques. http://www.insee.fr/en/default.asp

[ref-3139966594] Kusnik-Joinville O, Weill A, Ricordeau P, Allemand H. Diabète traité en France en 2007 : un taux de prévalence proche de 4% et des disparités géographiques croissantes. BEH 12 novembre 2008 / n° 43.

[ref-4254020960] Étude nationale nutrition santé, 2006. http://www.invs.sante.fr/publications/2007/nutrition_enns/RAPP_INST_ENNS_Web.pdf

[ref-4026039681] ObEpi-Roche 2009. http://www.roche.fr/gear/newcontents/servlet/staticfilesServlet?type=data&communityId=re719001&id=static/attachedfile/re7300002/re72700003/AttachedFile_10160.pdf

[ref-3159098297] Saudubray T, Saudubray C, Viboud , Jondeau G, Valleron AJ, Flahault A, Hanslik T. Prevalence and management of heart failure in France: national study among general practitioners of the Sentinelles network. Rev Med Interne 2005; 26: 845-50. 10.1016/j.revmed.2005.04.038 15935520

